# Concurrent Use of Mepolizumab and Rituximab for Eosinophilic Granulomatosis With Polyangiitis and Multisystem Involvement

**DOI:** 10.7759/cureus.9242

**Published:** 2020-07-17

**Authors:** Agura Afiari, Andre Gabriel, Meghana R Gaiki

**Affiliations:** 1 Internal Medicine, University of Connecticut (UCONN) School of Medicine, Hartford, USA; 2 Internal Medicine, University of Connecticut (UCONN) Health, Farmington, USA; 3 Nephrology, Saint Francis Hospital and Medical Center, Hartford, USA

**Keywords:** mepolizumab, egpa, rituximab, glomerulonephritis, vasculitis

## Abstract

Eosinophilic granulomatosis with polyangiitis (EGPA), formerly Churg-Strauss, is an anti-neutrophil cytoplasmic antibody (ANCA)-associated autoimmune vasculitis, involving small- and medium-sized arteries, which could involve several organs. This rare syndrome can present with a myriad of symptoms, which may make diagnosis challenging. It has been suggested that there are variants of EGPA, which may respond differently to available modes of treatment. Multiple and different mechanisms may be at play in each case of EGPA. This may influence the decision of clinicians to combine treatment strategies as done in this case. The addition of immunosuppressive agents other than high-dose steroids may mitigate end-organ damage, facilitate faster recovery, and prevent relapse. Rituximab among others has been seen to provide better outcomes, including a lower incidence of relapse. Mepolizumab was approved by the Food and Drug Administration (FDA) in 2017 for the treatment of EGPA. Administered at a higher dose than approved for severe eosinophilic asthma, it has been shown to lengthen remission in EGPA. The optimal dose and duration of therapy with mepolizumab remain unclear. The rarity alone of EGPA creates room for further investigation regarding pathogenesis, outcome over time, and treatment strategies, which may vary depending on how an individual case presents.

This case describes the course of a 55-year-old woman who presented with respiratory symptoms, pauci-immune necrotizing granulomatous nephropathy, and neuropathy secondary to P-ANCA-positive EGPA who was successfully treated with rituximab and mepolizumab, in addition to glucocorticoids.

## Introduction

Eosinophilic granulomatosis with polyangiitis (EGPA), formerly Churg-Strauss, is an anti-neutrophil cytoplasmic antibody (ANCA)-associated autoimmune vasculitis involving small- and medium-sized arteries. It was first described in 1949 in a group of patients with eosinophilia, asthma, fever, and vasculitis, which involved several organs [1]. For this reason, this syndrome can present with a myriad of symptoms that may make diagnosis challenging. The incidence of EGPA in the United States has been estimated at 1.3/100,000 [2]. With this knowledge, there is the concern that EGPA is underdiagnosed and there is limited understanding of its pathophysiology, hence, there is room to optimize current therapy or even discover new treatment strategies. It has been suggested that there are variants of EGPA that may respond differently to available modes of treatment. Immunosuppressive agents, including cyclophosphamide, azathioprine, mycophenolate mofetil, rituximab, and, more recently, mepolizumab, are shown to be effective in different treatment strategies.

The case below describes the course of a 55-year old woman who presented with respiratory symptoms, nephropathy, and, neuropathy secondary to P-ANCA-positive EGPA and was successfully treated with rituximab and mepolizumab, in addition to glucocorticoids.

## Case presentation

A 55-year old female with a past medical history of bilateral knee osteoarthritis, hypertension, and hypothyroidism presented to her primary care provider with joint pain and body aches for some months. She had visited the emergency department a few times for these symptoms and was managed with short courses of low-dose prednisone. outpatient lab work revealed C-reactive protein (CRP) and erythrocyte sedimentation rate (ESR) significantly elevated. Other immune markers, including anti-dsDNA, anti-Ro, anti-La, anti-Smith, RNP, RF, CCP, and HLA-B27, were found to be in the normal range. She was diagnosed with polymyalgia rheumatica and prescribed prednisone 15 mg to be taken daily.

Six months later, she returned to the primary care physician’s office with vague symptoms of low-grade fevers, dry cough, nasal and sinus congestion, paresthesia and numbness of her feet, malaise, and nausea. She stated that she had tapered down her prednisone unbeknownst to her rheumatologist. She denied nonsteroidal anti-inflammatory drugs (NSAIDs) use, new medications, poor oral intake, diarrhea, vomiting, rash, dysgeusia, and pruritus. She reported taking herbal supplements, including turmeric. She was on pantoprazole for gastroesophageal reflux disease.

**Figure 1:**
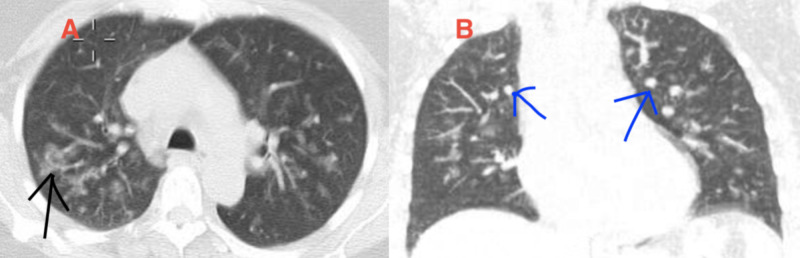
Multifocal nodular airspace opacities seen on chest CT Sub-panel A: axial view showing multifocal opacities (black arrow); Sub-panel B: coronal view showing multifocal nodular opacities (blue arrows) CT: computed tomography

**Figure 2:**
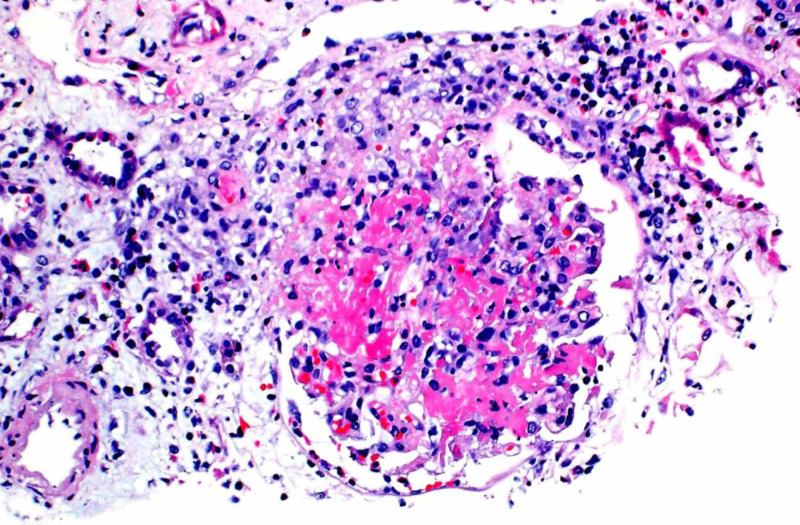
Necrotizing and crescentic glomerulonephritis

**Figure 3:**
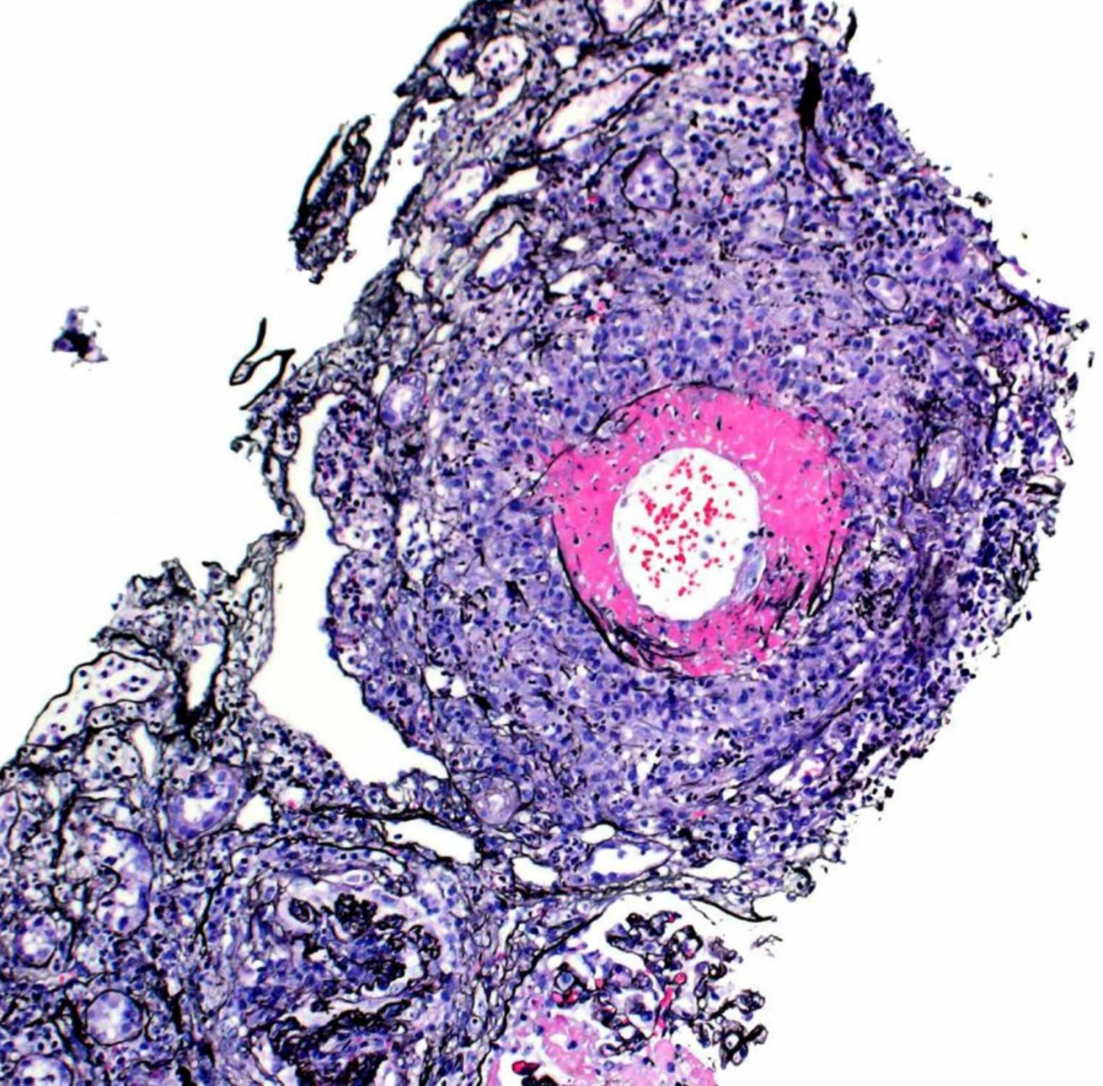
Necrotizing granulomatous arteritis

**Figure 4:**
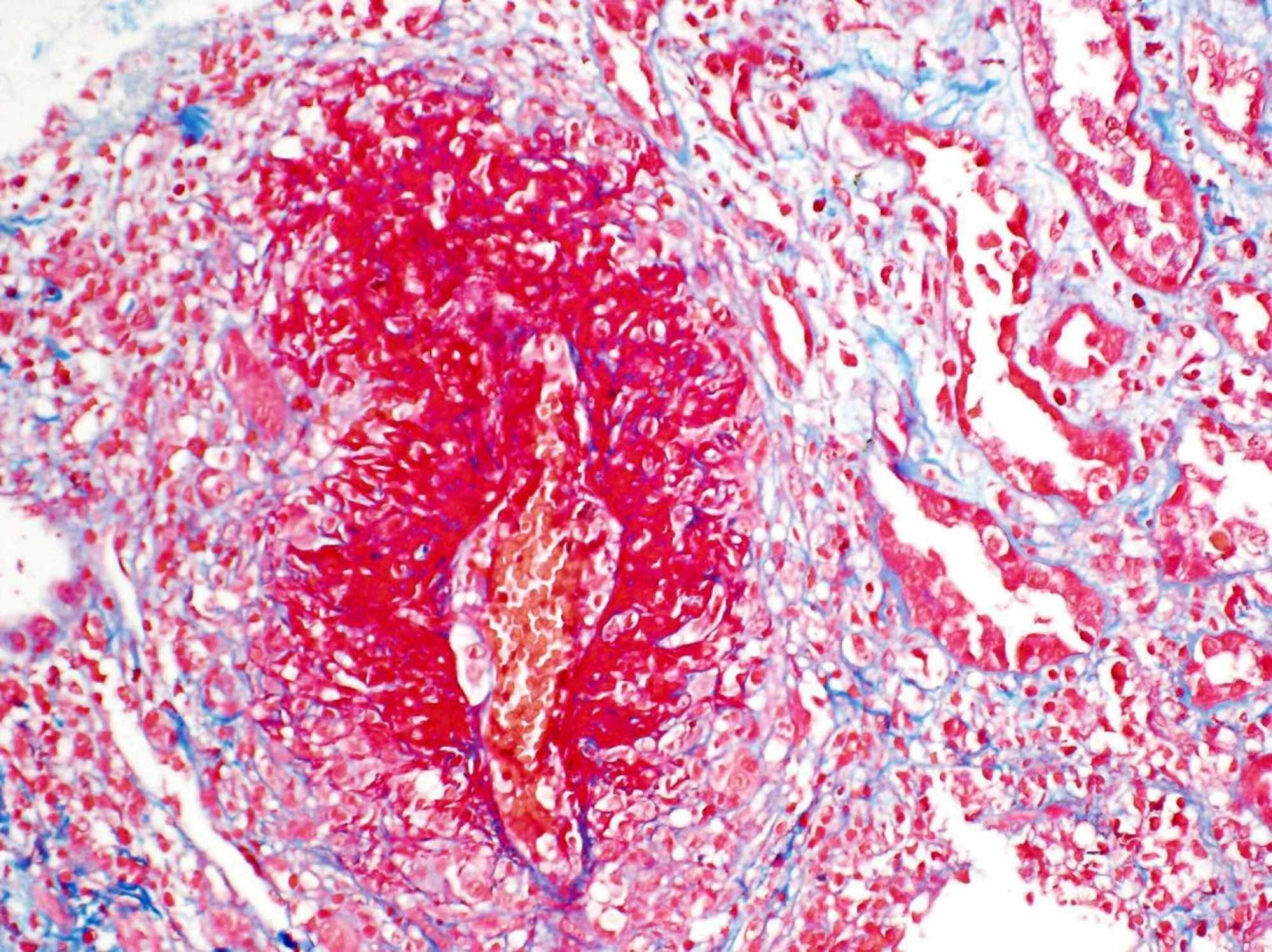
Necrotizing granulomatous arteritis

**Figure 5:**
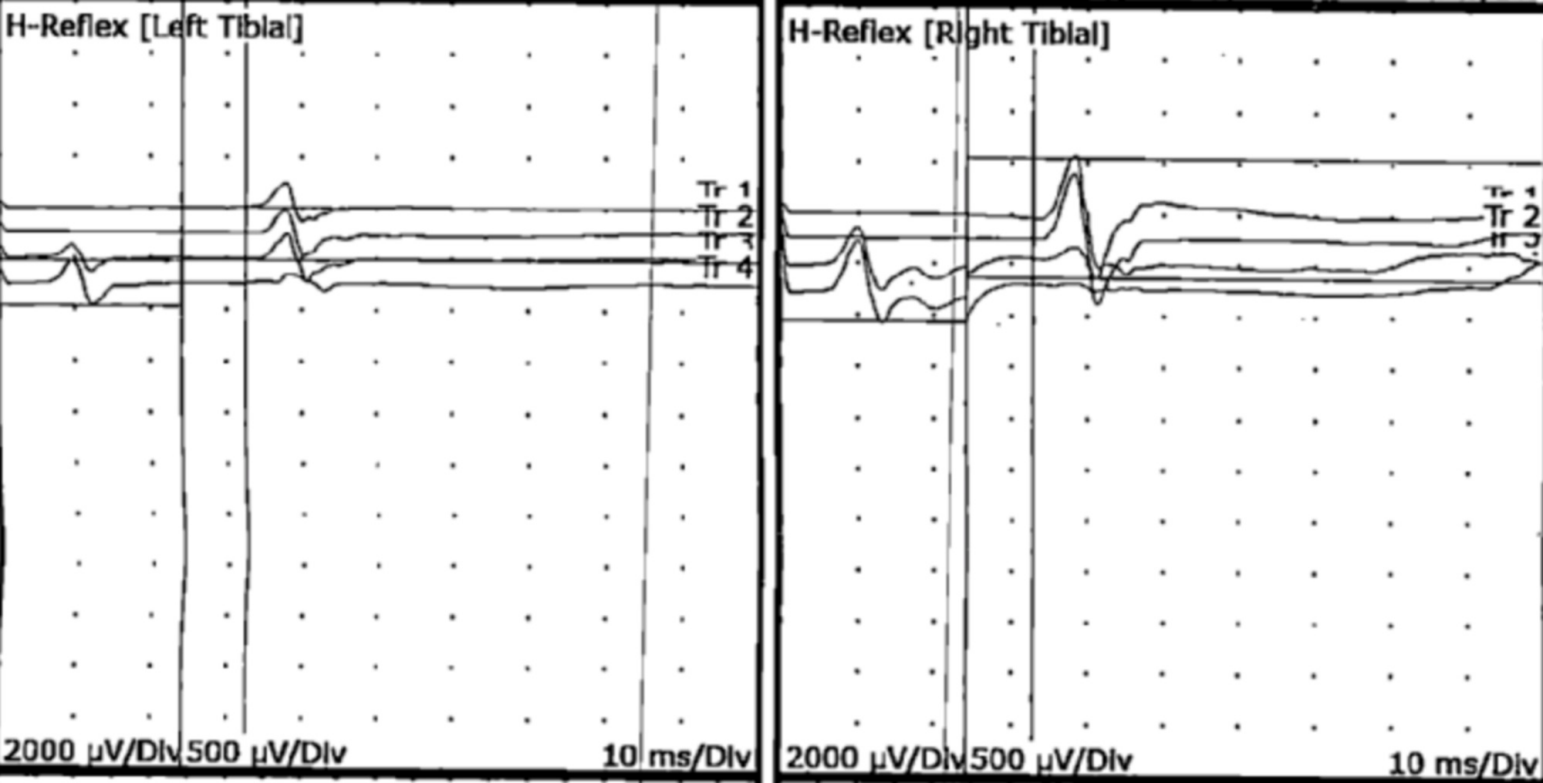
Motor nerve conduction studies of right and left tibial muscles

**Figure 6:**
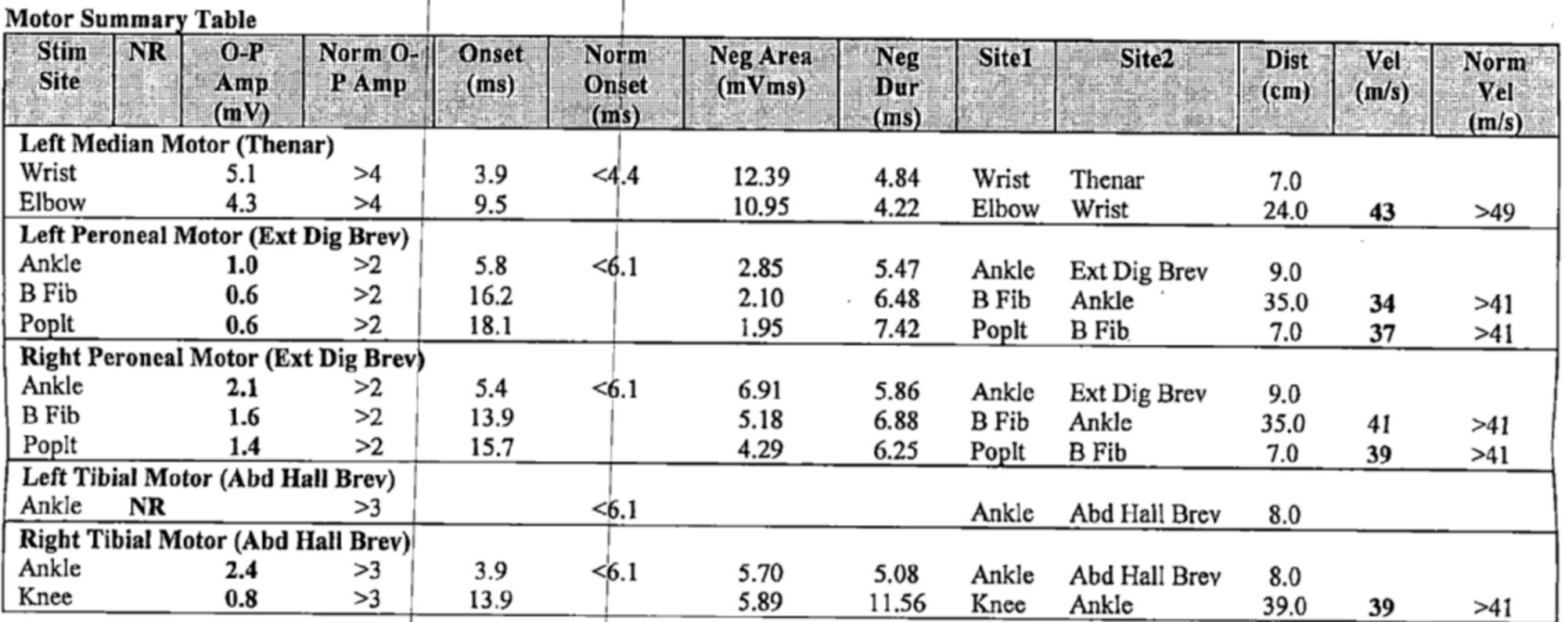
Motor nerve conduction studies summary Electrodiagnostic evidence of an asymmetric sensorimotor polyneuropathy (mild to moderate), primarily affecting the lower extremities (L>R), due to chronic axonal and secondary demyelinating nerve injury

**Figure 7:**
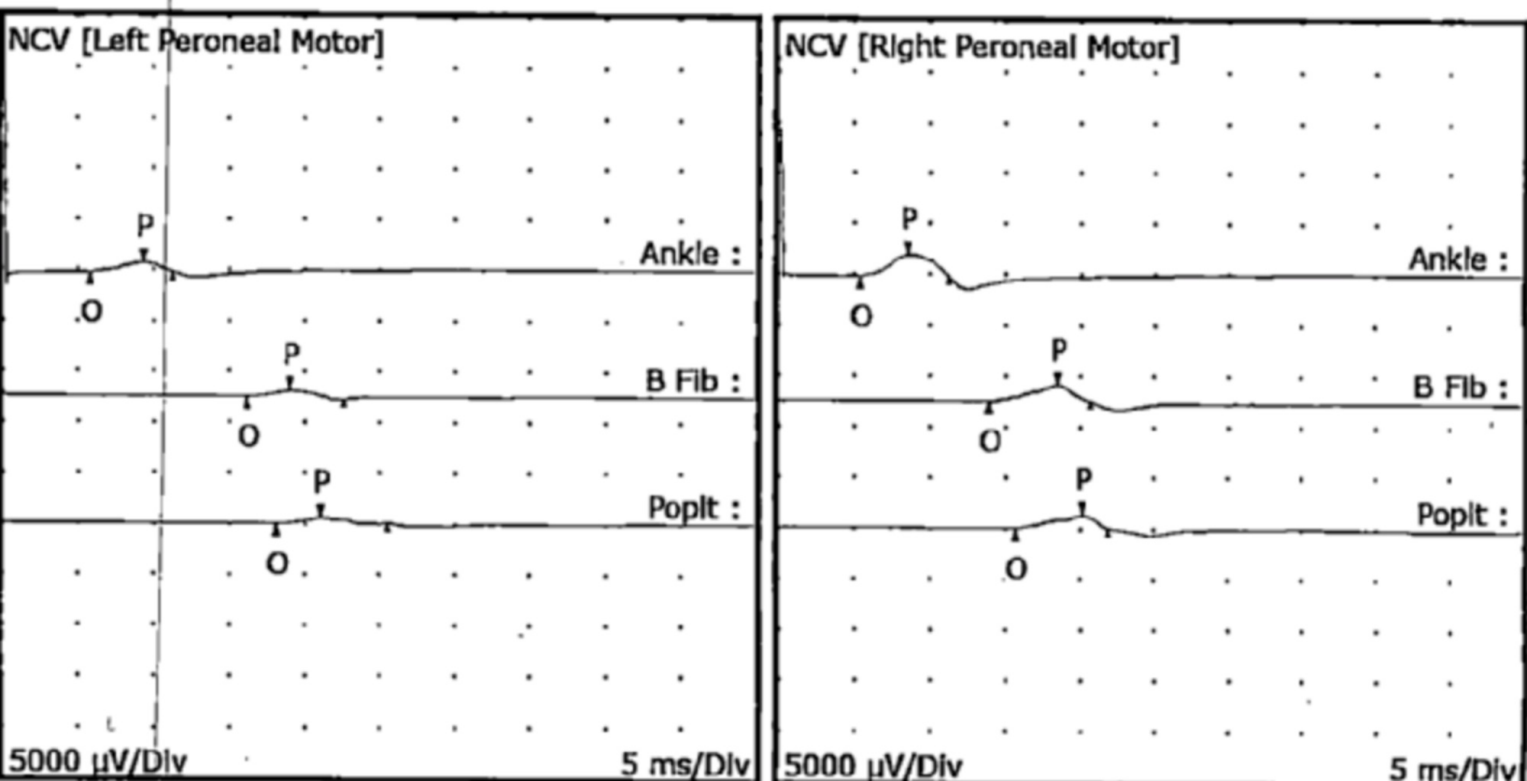
Left and right peroneal motor nerve conduction studies

Due to acute renal failure, seen in outpatient lab work, she was sent to the hospital for further evaluation. Vitals were stable on room air and her physical exam was unrevealing. Lab work was significant for blood urea nitrogen (BUN)/creatinine (Cr) 41/3.4 (baseline creatinine 0.8 a year prior), white blood cell (WBC) 14.6, eosinophils 3600 (normal 15-500 eos/microL). P-ANCA >1:320. Urine microscopy showed microscopic hematuria with both normal and dysmorphic red blood cells (RBCs). There were no casts and rare WBCs. The renal ultrasound (US) was unrevealing. A chest CT (Figure 1) was obtained that revealed multifocal nodular airspace opacities. She was started on pulse dose steroids. Renal biopsy revealed necrotizing and crescentic glomerulonephritis, (pauci-immune type), necrotizing granulomatous arteritis, and severe eosinophilic and neutrophilic tubulointerstitial infiltrate (Figures 2-4). Electromyography (EMG) showed asymmetric sensorimotor polyneuropathy affecting the lower extremities (L>R) due to chronic axonal and secondary demyelinating (mixed) nerve injury (Figures 5-7). The decision was made to place her on rituximab weekly for four doses and mepolizumab 300 mg monthly, which were started while inpatient. Steroid taper was continued and atovaquone was added for Pneumocystis jiroveci pneumonia (PJP) prophylaxis. She has been able to return to work full time. Two months after, her renal function stabilized with creatinine at 1.6 mg/dl, improvement in wheezing and congestion, occasional cough, and persistent left foot paresthesia.

## Discussion

Eosinophilic granulomatosis with polyangiitis (EGPA), formerly Churg-Strauss syndrome, is autoimmune vasculitis involving small- and medium-sized arteries, which could potentially lead to pathology in all organs. The typical presentation is a patient with upper and/or lower respiratory symptoms and further investigation leading to the detection of eosinophilia. As any organ system may be affected, the presentation may vary widely and may also be nonspecific. The above patient reported a history of allergies for years before her presentation. She ultimately presented with upper and lower respiratory symptoms, paresthesia, and numbness of feet confirmed as demyelinating polyneuropathy by electromyography (EMG) and renal failure (biopsy result above). P-ANCA being more common than C-ANCA, the incidence of ANCA-associated vasculitis is rare, with EGPA being the rarest of the three and the others being microscopic polyangiitis and granulomatosis with polyangiitis (Wegener’s). These are also to be considered in the evaluation of a patient with the pulmonary-renal syndrome.

Using both the American College of Rheumatology (ACR) [3] and Lanham [4] criteria, the above patient’s presentation was diagnostic for EGPA. The presence of extrapulmonary manifestation helps in narrowing the diagnosis, as other eosinophilic lung diseases may present similarly. Among others, acute interstitial nephritis secondary to drugs, infections, as part of autoimmune diseases (for example, immunoglobulin G4 (IgG4)-related kidney disease), and drug rash with eosinophilia and systemic symptoms (DRESS), are to be considered possible diagnoses in renal dysfunction with eosinophilia presentation.

The pathogenesis of EGPA is yet to be fully elucidated. A pauci-immune process is seen in the renal biopsy from the above case. This seems to be predominant in EGPA as opposed to other vasculitides where immune complex deposition has been described. Multiple and different mechanisms may be at play in each case of EGPA. Our patient had recently weaned herself to lower doses of prednisone and was seen to have a high P-ANCA titer of >1:320. These together may have played a role in the pathophysiology, specifically in this case. Comarmond et al. describe a different pattern of presentation in patients with P-ANCA positive vs P-ANCA negative EGPA [5]. A predominance of renal involvement and peripheral neuropathy in ANCA positivity has been demonstrated [6], which is seen in the above case.

We decided it was necessary to add on other immunosuppressive agents other than high-dose steroids because of the degree of multisystem involvement observed. This was to mitigate end-organ damage and facilitate faster recovery and prevention of relapse. Rituximab and mepolizumab were used with glucocorticoids for both induction and maintenance therapy. This may have been of added benefit considering likely multiple players in the pathogenesis of EGPA. The mainstay of treatment is immunosuppression with corticosteroids and other immunomodulators. Such drugs, including cyclophosphamide, mycophenolate mofetil, azathioprine, and rituximab among others, in addition to steroid chronic therapy, have been seen to provide better outcomes including a lower incidence of relapse. Thiel et al. demonstrated rituximab as an effective therapy for induction remission even in EGPA patients who had been refractory to standard therapy, including cyclophosphamide [7]. It may also be more beneficial to ANCA-positive patients [8]. The side-effect profile of each immunosuppressive agent must be considered per patient when determining which is most suitable.

Mepolizumab was approved by FDA in 2017 for the treatment of EGPA. It is a monoclonal antibody that affects the pathway leading to eosinophil maturation and activation via interleukin 5 antagonism. Concurrent use of mepolizumab and rituximab (destruction of CD20+ B lymphocytes) for our patient will target different pathways of the immune system involved in eosinophil activation, which, in theory, can produce a synergistic effect in the treatment of EGPA compared to the use of each agent alone. Mepolizumab has been shown to provide longer periods of remission [9]. For this reason, a randomized control trial by Wechsler et al. used 300 mg of mepolizumab administered subcutaneously every four weeks (compared to 100 mg used in severe eosinophilic asthma), which showed longer remission and lower rates of relapse in patients with EGPA refractory to other modes of therapy [10]. In a case report, 100 mg of mepolizumab was successfully used for maintenance therapy after induction with rituximab in a patient who presented with diffuse alveolar injury in the setting of cyclophosphamide refractory EGPA [11]. This raises the question of what the optimal dose for EGPA is. Also, the optimal duration of therapy with mepolizumab remains unclear, as cases of relapse after discontinuation have been documented. Other studies have described the possible benefit of plasma exchange [12] and renal transplantation [13] in patients with more severe renal dysfunction and ESRD, respectively.

## Conclusions

The rarity of EGPA alone creates room for further investigation regarding pathogenesis, outcome over time, and treatment strategies. The possibility of several mechanisms at play in the pathogenesis may mean that different treatment options may be required depending on how each case presents. The presence or absence of P-ANCA, what organ system is involved, the severity of the illness, the age of the patient, may all need to be considered when determining treatment modalities. The observed reduction in mortality rates from earlier suggests that some progress has been made so far. In this case, a biopsy of the kidney early in the disease process provided the benefit of a clearer diagnosis and, hence, better targeted, and early treatment, preventing progression to more severe renal injury.
